# Effect of Interactions on the Nutrient Status of a Tropical Soil Treated with Green Manures and Inorganic Phosphate Fertilizers

**DOI:** 10.1100/tsw.2004.35

**Published:** 2004-06-08

**Authors:** Abdul R. Bah, Zaharah A. Rahman, Aminuddin Hussin

**Affiliations:** Department of Land Management, Universiti Putra Malaysia, 43400 Serdang, Selangor Darul Ehsan, Malaysia

**Keywords:** acid soil infertility, ammonium-N, available P, exchangeable cations, green manures, integrated nutrient management, nutrient uptake, phosphate rock, P retention, 33P-32P double isotopic labeling techniques, pH, soil P fractions

## Abstract

Integrated nutrient management systems using plant residues and inorganic P fertilizers have high potential for increasing crop production and ensuring sustainability in the tropics, but their adoption requires in-depth understanding of nutrient dynamics in such systems. This was examined in a highly weathered tropical soil treated with green manures (GMs) and P fertilizers in two experiments conducted in the laboratory and glasshouse. The treatments were factorial combinations of the GMs (*Calopogonium caeruleum, Gliricidia sepium*, and *Imperata cylindrica*) and P fertilizers (phosphate rocks [PRs] from North Carolina, China, and Algeria, and triple superphosphate) replicated thrice. Olsen P, mineral N, pH, and exchangeable K, Ca, and Mg were monitored in a laboratory incubation study for 16 months. The change in soil P fractions and available P was also determined at the end of the study. Phosphorus available from the amendments was quantified at monthly intervals for 5 months by P-P double isotopic labeling in the glasshouse using *Setaria sphacelata* as test crop. The GMs were labeled with P to determine their contribution to P taken up by *Setaria*, while that from the P fertilizers was indirectly measured by labeling the soil with P. The P fertilizers hardly changed Olsen P and exchangeable cations during 16 months of incubation. The legume GMs and legume GM+P did not change Olsen P, lowered exchangeable Ca, and increased exchangeable K about threefold (4.5 cmol[+]kg soil) in the first 4 months, even as large amounts of NH_4_-N accumulated (~1000 mg kg soil) and soil pH increased to more than 6.5. Afterwards, Olsen P and exchangeable Ca and Mg increased (threefold) as NH_4_-N and soil pH declined. The legume GMs also augmented reversibly sorbed P in Al-P and Fe-P fractions resulting in high residual effect in the soil, while fertilizer-P was irreversibly retained. The GMs increased PR-P utilization by 40 to over 80%, mobilized soil P, and markedly enhanced uptake of N, K, Ca, and Mg. Thus GMs+PRs is an appropriate combination for correcting nutrient deficiencies in tropical soils.

### INTRODUCTION

Increasing crop production in many areas of the tropics (especially sub-Saharan Africa) is faced with major challenges because of the predominance of highly weathered and inherently infertile Ultisols and Oxisols[1]. These soils have severe chemical constraints associated with low pH including low cation saturation, toxic concentrations of Al, as well as deficiency in N and P[2]. Other factors include declining arable land area due to soil degradation and demographic pressure[3], and the inadequate use of high analysis fertilizers because of severe socioeconomic constraints on the farmers. Intensifying crop production in these circumstances often results in depletion of organic matter[4], high nutrient losses[5], and ultimately severe land degradation[6]. Consequently, soil fertility restoration and maintenance programs in the tropics need to emphasize the judicious use of chemical fertilizers coupled with improved management of soil organic matter to enhance nutrient cycling, mitigate losses, and hence maintain environmental quality. This could be through improved fallow management with N-fixing trees, hedgerow intercropping with legumes, biomass transfer, and the use of organic inputs like green manures (GMs) and animal manures[7].

Decomposing organic materials generally release N, P, and basic cations concomitantly with organic anions, which can detoxify heavy metal ions through complex formation[8]. Therefore, they have high potential for improving tropical soils. However, while crop N requirements could be satisfied easily by GM applications, that of P are fraught with major problems because of the generally low P content of organic matter[9]. This implies enormous labor requirements for the management of P using organic materials because of their scarcity and bulky nature.

A better alternative for improving productivity in tropical soils would be to integrate organic materials with cheaper inorganic P sources such as phosphate rocks (PRs)[10,11]. This is a more attractive and sustainable option for resource-poor farmers who cannot afford costly, manufactured fertilizers. The combined use of GMs and PRs has been shown to enhance the utilization of PR-P in acid soils[10,12]. In principle, the degrading GMs and PRs in the combined system release nutrients that can create conditions that would either promote or adversely affect soil nutrient status. For instance, both materials release Ca, which in the absence of a sink can retard further P release from PRs[13]. Similarly, GM decomposition increases soil pH[8], which at moderate levels facilitates P desorption[14], but can exacerbate P retention at higher levels[15]. At the same time, soil microbial activity is promoted and significant amounts of nutrients can be immobilized in the microbial biomass[16]. All these processes would have significant impact on plant-available nutrients in the soil. Therefore, there is need for better understanding of nutrient interactions and secondary transformations that occur when PRs and GMs are incorporated together in tropical soils. This is important for adapting such integrated nutrient management systems to various cropping systems in the tropics. As a result, this study was carried out to evaluate the effect of PRs and GMs on nutrient dynamics and soil nutrient status, and to demonstrate the potential of such a system for enhancing productivity in a tropical soil. Some emphasis was made on available P dynamics because P is the most limiting as well as the most difficult nutrient to replenish in tropical soils. Thus the influence of GMs on the efficacy of reactive and poorly reactive PRs in increasing plant-available P in this soil was investigated using P tracer techniques. In this regard, the contribution of the GMs was assessed by directly labeling each of them with $^{33}$P radioisotope. Labeling PRs without altering their characteristics is difficult, so the contribution of the PRs to P availability was indirectly determined by the $^{32}$P isotope dilution technique. The latter is based on the principle that P tracers undergo exchange reactions with chemically identical, but isotopically different forms of P in a soil system. This results in the dilution of the P tracer, so available P contribution from PRs is determined by assuming that when a plant has more than one nutrient source, the quantity taken up from each source will be in direct proportion to its availability. Thus their contributions to plant-available P can be obtained without disturbing the soil system.

### MATERIALS AND METHODS

Laboratory incubation and glasshouse studies were carried out with a tropical soil treated with factorial combinations of three GMs and four P fertilizers. The soil was topsoil (0–20 cm) from the Bungor series

(Typic Paleudult, kaolinitic, isohyperthermic) with pH water (1:2.5) of 4.8 and 1 kg soil has 18 g organic C, 120 mg N, 10.9 mg Olsen P, 8 g $Al_2O_3$, 24 g $Fe_2O_3$, 275 g clay, and 559 g sand. Also, cation exchange capacity (CEC) is 5.4 cmol (+) $kg^{-1}$ with 0.37, 0.92, 0.44, and 1.8 cmol (+) $kg^{-1}$ exchangeable K, Ca, Mg, and Al, respectively. Soil pH was measured in distilled water (1:2.5 w/v) using a glass electrode, organic C by the dichromate wet oxidation method[17], total N by the Kjeldahl method[18], and available P by extraction with 0.5*M* $NaHCO_3$ at pH 8.2[19] and then measuring P in the extract by the method of Murphy and Riley[20]. Active Al and Fe were analyzed by dithionite extraction[21] followed by measurement with an atomic absorption spectrophotometer. Particle size analysis was carried out by the pipette method after removing organic matter with $H_2O_2$ and dispersing soil particles with sodium hexametaphosphate. Also, exchangeable acidity was assessed by extraction with 1*M* KCl (1:5, w/v) followed by titration with NaOH. Exchangeable Al in this extract was determined by atomic absorption spectrophotometry. CEC and exchangeable K, Ca, and Mg were measured by first leaching the soil with neutral molar $NH_4OAc$ to saturate exchange sites with $NH_4^+$ and leach out adsorbed cations, then eluting the adsorbed $NH_4^+$ with 0.5*M* $K_2SO_4$. The exchangeable cations were analyzed in the first leachate by atomic absorption spectrophotometry following dilution of an aliquot of the leachate with 1000 μg $ml^{-1}$ $SrCl_2$. The CEC was estimated in the second leachate via steam distillation wherein the distillate was collected in 2% $H_3BO_3$ and titrated with 10 *mM* HCl. The GMs were green leaves of *Gliricidia sepium* (Gliricidia) and *Imperata cylindirica* (Imperata), and the leaves plus green stems of *Calopogonium caeruleum* (Calopogonium) (Table 1). Chemical analysis of the GMs was conducted on oven-dried (70°C), ground (<1 mm) samples. Total P, K, Ca, and Mg were measured after ashing at 550°C for 4 h. The ash was dissolved in 2*M* HCl and evaporated to dryness. The residue was then dissolved in 20% $HNO_3$ and analysis of the elements carried out as described above. Total N was also measured as in the soil. Total polyphenols was determined by the Folin-Denis procedure and lignin by the acid detergent fiber method[22]. For the glasshouse study, the GMs were labeled with $^{33}P$ by applying a solution of $^{33}P$ (activity 3.33 MBq) and 18 μg P ($KH_2PO_4$) carrier to 15-month-old plants grown in Al containers. The plants were allowed to grow for another 3 weeks before harvesting the GMs. The P fertilizers were triple superphosphate (TSP) and PRs from Algeria (APR), China (CPR), and North Carolina (NCPR) (Table 2). The P fertilizers were characterized in terms of both elemental content and their solubility in citric acid. Two grams of each P fertilizer was digested by adding subsamples to a mixture of $HNO_3$ and HCl (4 ml each), in 50 ml $H_2O$ and boiling for 30 min. After filtration, determinations were carried out for P, Ca, Fe, and Al as described above. The solubility test was carried out with 2% citric acid (1:100, w/v).

### Incubation Procedure

One kilogram of air-dried soil was sieved (<2 mm) and the moisture content increased to 90% of field capacity with distilled water in plastic bags to stimulate microbial activity. These were put aside for 14 d for equilibrium to be established. Factorial combinations of the fresh GMs (5% w/w, dry weight basis) and P fertilizers (66. 7 mg P $kg^{-1}$ soil) were then mixed thoroughly with the soil in three replications and completely randomized. An untreated control was also added. Imperata and Calopogonium were cut into smaller pieces (1–2 cm) to minimize size differences among the GMs. Soil samples were collected at weekly intervals in the first month and thereafter at 2, 4, 8, and 16 months after treatment application, to measure $NH_4$-N and $NO_3$-N[23], pH, exchangeable K, Ca, and Mg by the neutral molar $NH_4OAc$ method and bicarbonate extractable P[19]. The final samples were also sequentially extracted for P fractions[19,24] and organic P was also determined[25]. Plant-available P was estimated by the iron oxide impregnated paper strip (Pi) method[26].

**TABLE 1**
**Biochemical Characteristics of the GMs Used**

| Parameter (g $kg^{-1}$) | Gliricidia | Imperata | Calopogonium |
|---|---|---|---|
| N | 41.1 | 12.6 | 32.3 |
| P | 1.8 | 0.8 | 2.6 |
| C | 412.0 | 400.0 | 388.0 |
| Ca | 5.4 | 0.9 | 3.3 |
| Mg | 3.6 | 1.1 | 1.6 |
| K | 24.4 | 12.4 | 25.0 |
| Lignin (Lig) | 128.6 | 129.9 | 135.0 |
| Polyphenols (Poly) | 14.7 | 27.2 | 8.8 |
| C:N | 10 | 32 | 12 |
| C:P | 229 | 500 | 149 |
| (Lig+Poly):N | 3.5 | 12.5 | 4.4 |

**TABLE 2**
**Chemical Properties of the P Fertilizers Used**

| Composition (%) | P Fertilizers | | | |
|---|---|---|---|---|
| | TSP | APR | NCPR | CPR |
| $P_2O_5$ | 46.24 | 30.07 | 30.90 | 31.95 |
| CaO | 19.32 | 46.54 | 45.03 | 42.27 |
| $CaO/P_2O_5$ | 0.42 | 1.55 | 1.46 | 1.32 |
| $Fe_2O_3$ | 0.79 | 0.09 | 0.28 | 0.24 |
| $Al_2O_3$ | 3.21 | 0.79 | 0.89 | 0.89 |
| Solubility* | 43.75 | 36.91 | 23.92 | 10.58 |

* 2% citric acid solubility expressed as % of total P.

### Glasshouse Studies

The same treatments were used here except that the soil was labeled with a mixture of $^{32}P$ (1.85 MBq) and 2 mg P carrier ($KH_2PO_4$) before being set aside for equilibrium to be established. The pool of available P in the soil becomes labeled with $^{32}P$ through exchange reactions. This is "diluted" by the P contributed by the applied materials (GMs and P fertilizers) after treatment application. Thus plants grown in pots without treatment would show maximum uptake of $^{32}P$, which is reduced by the proportion of P contributed from the GMs and/or P fertilizers in the treated pots. In addition, the GMs were labeled with $^{33}P$ and this would give the amount of P directly contributed by the GMs. Two stem cuttings (12–15 cm) of *Setaria sphacelata* were planted in each pot and supplementary N and K (33.3 mg $pot^{-1}$) were added to treatments without GMs. The plants were manually irrigated with distilled water daily and harvested at 4, 8, 15, and 20 weeks after treatment application. Dry weight was recorded after oven drying to constant weight (70°C) and manually crushed subsamples were ashed at 550°C for 4 h. The ash was dissolved in 2*M* HCl, filtered, and the activities of $^{33}P$ and $^{32}P$ determined by liquid scintillation counting using the external standard channels ratio method to determine efficiency[27], and total P was measured by the Scheel[28] procedure. Total K, Ca, and Mg were also measured as described earlier. Percent P taken up from the GMs, %PdfGM, was then calculated from the following relationship:

$$\%PdfGM = (SA_{trt}/SA_{gm}) \times 100 \quad (1)$$

where $SA_{trt}$ is the specific activity of plants in the treated soil and $SA_{gm}$ is the specific activity of the applied GM.

$$\text{Total GM-P (mg pot}^{-1}) = \%\text{PdfGM} \times \text{PYLD}/100 \quad (2)$$

wherein PYLD is total P uptake (mg pot$^{-1}$), and

$$\text{PYLD} = \%\text{P} \times \text{DMY} \times 10 \quad (3)$$

DMY= dry matter yield (g pot$^{-1}$). Percent P derived from the fertilizer treatments (%PdfT) is given by the following equation

$$\%\text{PdfT} = \{1 - (SA_{amend}/SA_{control})\} \times 100 \quad (4)$$

where $SA_{amend}$ = specific activity of plants in the amended soil and $SA_{control}$ = specific activity of plants in the control.

### Data Analysis

Changes in the parameters monitored were calculated as differences between the treated soil and the control. Main effects of treatments were ascertained by analysis of variance (ANOVA) using the PROC GLM procedure of the SAS statistical package[29]. In cases where GM×P interactions were significant, the PROC SORT procedure was used to ascertain the effect of GMs on the P fertilizers for the parameters. Means separation was carried out by the least significant difference (LSD) method at $\alpha = 0.05$. Pearson's correlation analysis was used to ascertain relationships between some of the properties monitored and the contributions of soil P fractions to plant available P was determined by step-wise multiple regression.

## RESULTS AND DISCUSSION

### Effect of GMs and P Fertilizers on Soil Nutrients

The P fertilizers alone hardly affected soil mineral N, pH, and exchangeable cations due to the absence of N and low net alkalinity of these materials, as well as the buffering action of the soil. Also, less than 35% of applied P was available even from water soluble TSP and reactive APR due to retention of released P (large concentration of Al and Fe sesquioxides). Similarly, the legume GMs, alone or combined with inorganic P, had very little effect on available P, exchangeable Ca and Mg cations during the first 16 weeks (4 months), but markedly increased $NH_4$-N as well as soil pH during this period. Calopogonium had the greatest effect, 600 µg $NH_4$-N g$^{-1}$ (≈40% of added GM-N) was accumulated and pH increased from 4.8 to more than 6.5 in the first 8 weeks of the incubation (Figs. 1 and 2). This is consistent with the rapid release of N by high-quality GMs (low C/N, low lignin and polyphenols)[30,31]. Therefore, the very small buildup of $NH_4$-N in the Gliricidia treatment (<2% of applied GM-N) is rather surprising (C:N = 10; [lig + poly]:N = 3.5). This was likely due to volatilization of $NH_3$ owing to the low soil CEC, and perhaps the 5% application rate was too high for this GM[32]. As anticipated from its poor quality (C:N = 32; [lig + poly]:N = 12.5), Imperata caused little change in soil $NH_4$-N. The pH increases caused by GMs is generally attributed to ammonification, ligand exchange reactions between hydroxyl groups on soil surfaces and organic anions, as well as dissociation/association reactions of various organic acids and decarboxylation of organic anions by microbial action[8,33,34]. Ammonification was likely the

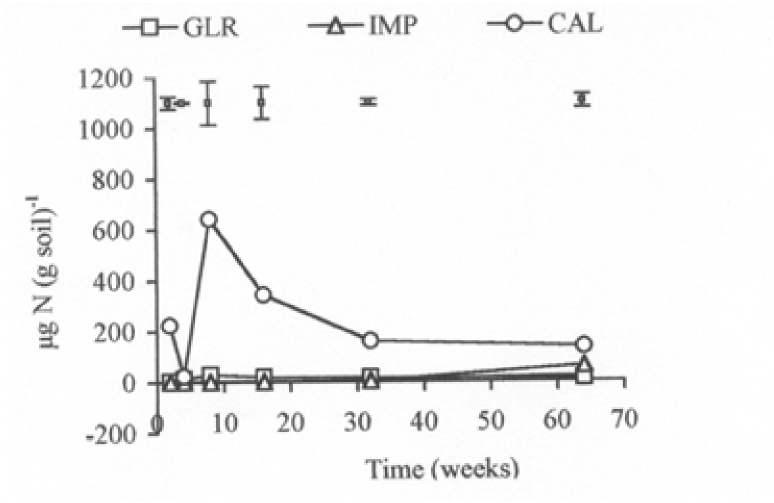

**Figure 1.** $NH_4$-N accumulation in a tropical soil treated with GMs. GLR = Gliricidia, IMP = Imperata and CAL = Calopogonium. Bars = $LSD_{0.05}$.

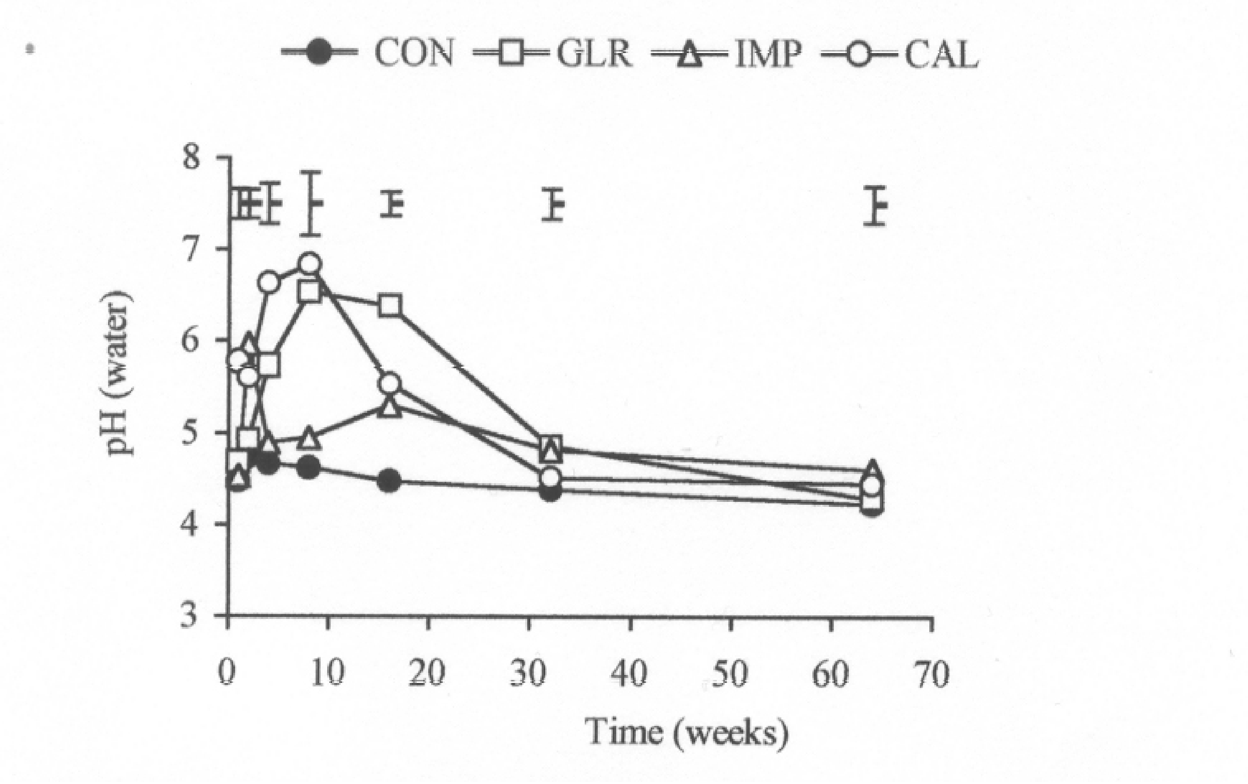

**FIGURE 2.** Changes in soil pH during incubation of a tropical soil treated with GMs.

overriding influence in our study as evidenced in the highly significant positive correlation between $NH_4$-N and soil pH in the early stages of the incubation (Table 3). The other mechanisms could have been important in the case of Gliricidia, considering that $NH_4$-N accumulation was very low in this treatment. The correlation diminished noticeably from 16 weeks onwards in line with the decline in soil $NH_4$-N probably due to loss through volatilization, nitrification, and depletion of organic N in the GMs during decomposition[30,35]. This coincided with the lowering of soil pH and remarkable increases in available P as well as exchangeable Ca and Mg (Figs. 3, 4, and 5).

**TABLE 3**
**Correlation Between pH and Some Soil Properties Monitored During Incubation of a Tropical Soil Treated with GMs Alone or in Combination with Inorganic P Fertilizers**

| Soil Property | Sampling Times (weeks) | | | | | |
|---|---|---|---|---|---|---|
| | 2 | 4 | 8 | 16 | 32 | 64 |
| $NH_4^+$-N | 0.73*** (n = 39) | 0.95*** (n =58) | 0.50*** (n = 45) | 0.13 ns (n = 48) | –0.01 ns (n = 42) | –0.18 ns (n = 27) |
| Exch. Ca | — | 0.44*** (n = 56) | –0.35** (n = 54) | 0.28* (n = 59) | 0.20 ns (n = 52) | — |

*, **, and *** denote significance at 5%, 1%, and 0.1% levels; n = sample size.

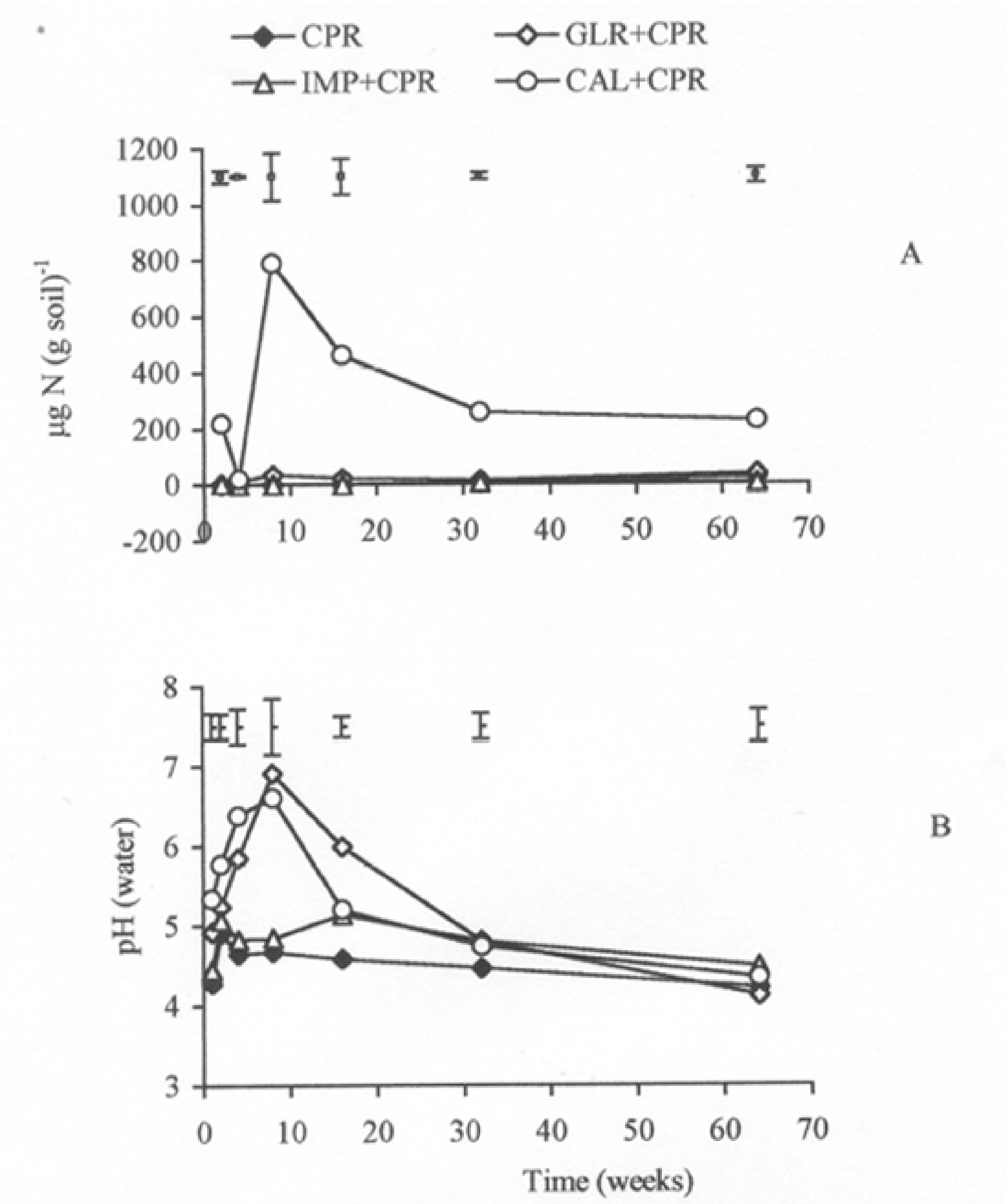

**FIGURE 3.** $NH_4$-N accumulation (A) and pH changes (B) in a tropical soil treated with GMs and PRs. CPR = China PR; bars = $LSD_{0.05}$.

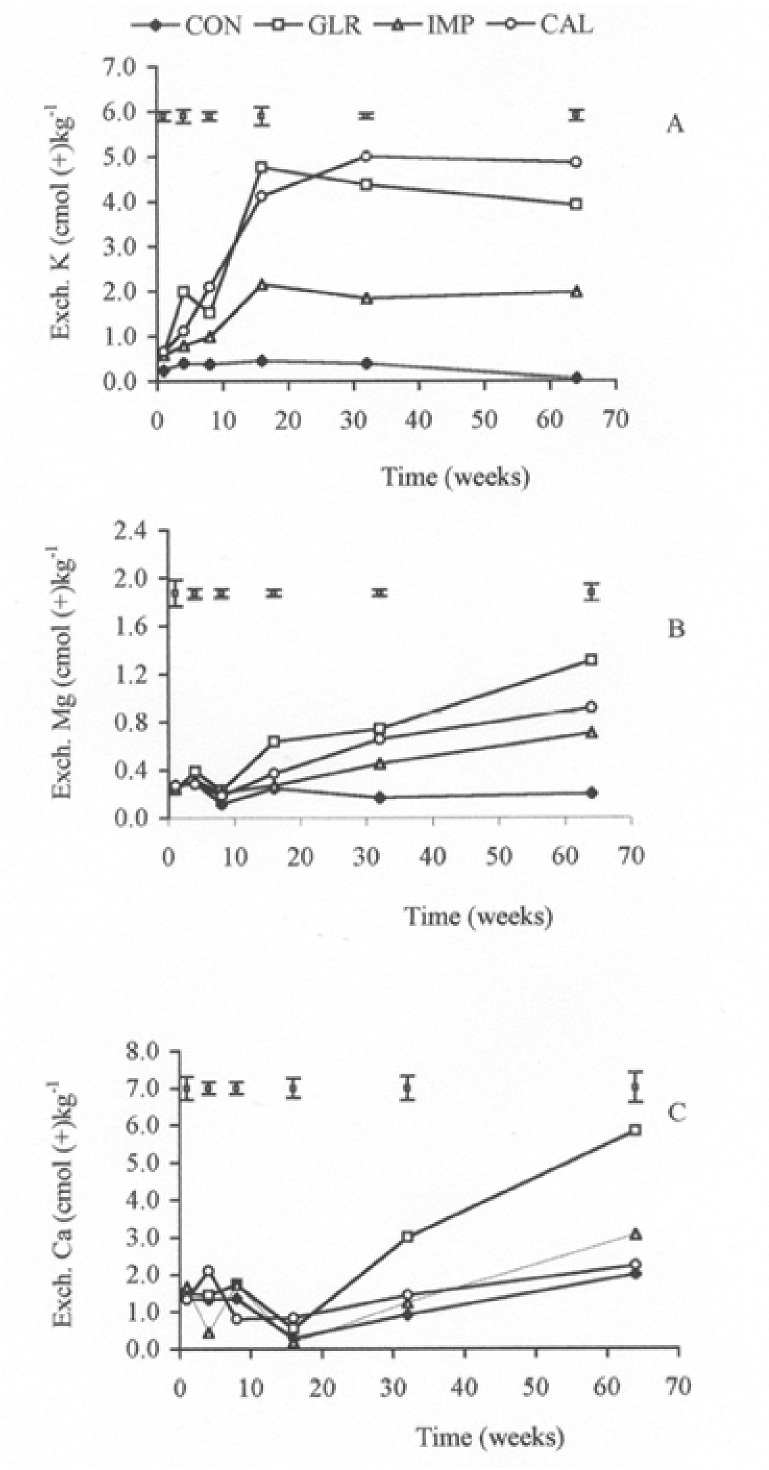

**FIGURE 4.** Changes in (A) exchangeable K, (B) Mg, and (C) Ca in a tropical soil treated with GMs and PRs. Bars = $LSD_{0.05}$.

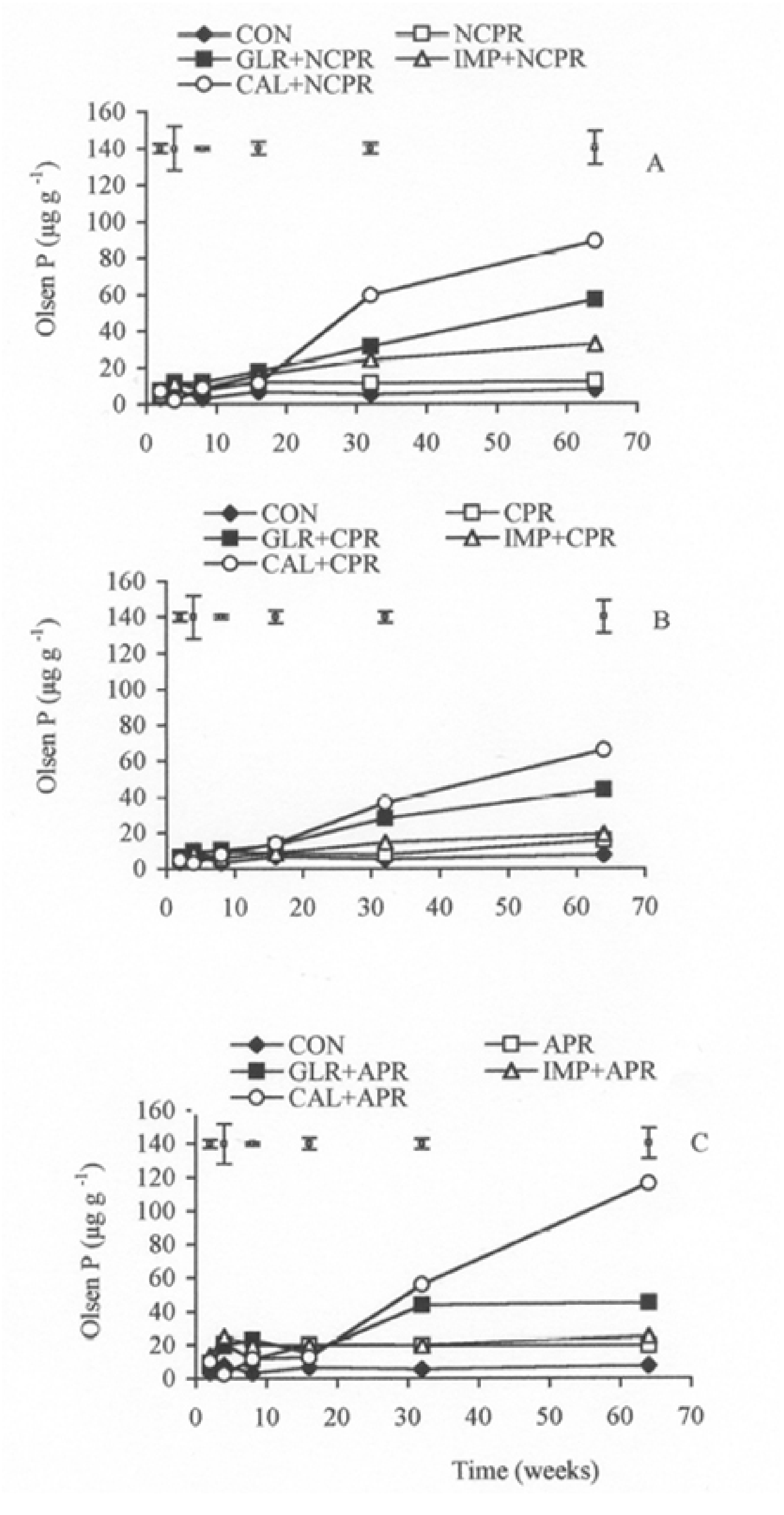

**FIGURE 5.** Changes in Olsen P in a tropical soil treated with GMs and PRs. (A) NCPR = North Carolina PR; (B) CPR = China PR; (C) APR = Algerian PR. Bars = $LSD_{0.05}$.

### Exchangeable Cations

Exchangeable Ca was generally low during the first 8–16 weeks of the incubation, while exchangeable K and Mg declined throughout the study period in soils treated with inorganic P fertilizers alone (data not shown). On the other hand, the decomposing GMs markedly increased exchangeable Ca after 16 weeks, and for K and Mg after the 8th week of incubation (Fig. 4). The extent of the effect was dependent on GM chemical characteristics (concentration of N, lignin and polyphenols, C/N, C/P; and concentration of K, Ca, and Mg), which govern their rate of decomposition as well as nutrient release patterns. Thus, the legume GMs generally caused much higher increases in exchangeable K and Mg than Imperata. As anticipated, Gliricidia (being the GM with the highest Ca content [Table 1]) caused the most significant buildup of exchangeable Ca beyond the 16$^{th}$ week of incubation, while the effect of Calopogonium was comparable with that of Imperata (Fig. 4). The pattern of change was different among the nutrients; exchangeable K increased to a maximum in 4 months (16 weeks) and there was little change thereafter. Calcium showed depressed levels during this period, but an increasing trend afterwards. Magnesium, on the other hand, increased gradually throughout the incubation study (Fig. 4). Beyond the 16$^{th}$ week, Gliricidia gave much higher Ca and Mg levels, while Calopogonium gave the highest soil K after 8 months. Imperata had the least effect on soil K and Mg, but showed comparable buildup of Ca with the Calopogonium treatment after the 4$^{th}$ month.

### Available P

The pattern of P availability was generally influenced by the release of other nutrients in the GM and GM+P treated soils. Ammonification resulted in the elevation of pH to more than 6.5 (Figs. 1–3), which increased P retention due to the precipitation of amorphous hydroxy Al polymers that acted as additional sorption sites[15,36]. Therefore, Olsen P was depressed in the GM- and GM+P-treated soil in the first 16 weeks of the incubation, and on average, less than 10% of the GM-P applied was available during this period (lag phase) (Figs. 5 and 6). This was most likely due to the high P retention capacity of the soil because of high sesquioxides, high Al saturation, and its 1:1 clay mineralogy[13]. Phosphorus retention in these conditions is via precipitation by Al and Fe ions, as well as adsorption on oxides and/or hydroxides of Al and Fe in the soil. This is evidenced by the significant increase in the Al-P and Fe-P fractions in the soil (Fig. 7). These P fractions have been associated with P sorption sites in weathered soils[8,14], which implies that the released P mainly saturated sorption sites. This confirms the central role of $PO_4$ ions in mitigating P retention. Also divalent cations such as $Ca^{2+}$ and $Mg^{2+}$ were released during GM decomposition[37]. These cations reduced the mobilization of adsorbed $PO_4^{3-}$ by converting it to less soluble phosphate compounds of Ca and Mg[38], and probably also immobilized available P in complex compounds like struvite, $NH_4MgPO_4.6H_2O$[39]. The Ca (from degrading PRs and GMs) could also suppress further P release from the PRs[13]. However, the low exchangeable Ca observed during the early phase of the incubation suggests a sink for the released Ca, probably through the formation of soluble complexes with organic anions. Perhaps this partly explains the lower exchangeable Ca values observed in this phase (Fig. 4). The precipitation of calcium phosphates could not have been one of the pathways for lowering exchangeable Ca and available P because the pH increase was not greater than 7[40]. Another pathway could have been through microbial action. It is known that the supply of soluble C by the high quality GMs fuels microbial activity, which results in the immobilization of considerable quantities of nutrients (including P) in the microbial biomass[41].

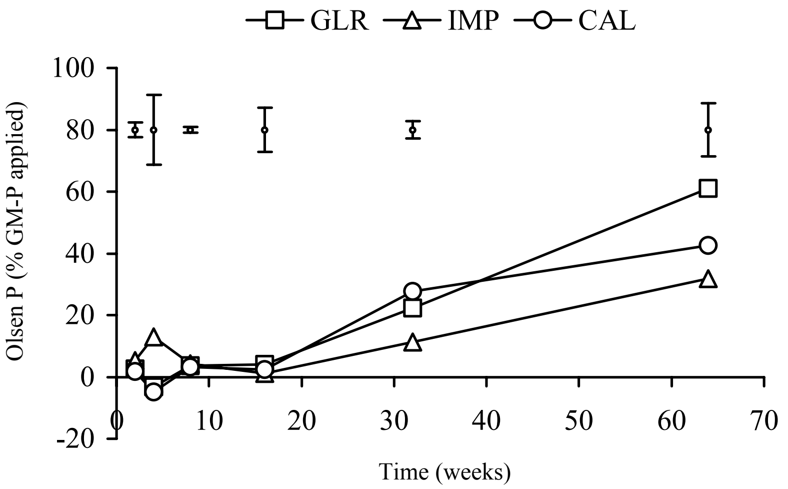

**FIGURE 6.** Net changes in Olsen P (as %GM-P applied) during incubation of a tropical soil treated with GMs. Bars = LSD $_{0.05}$.

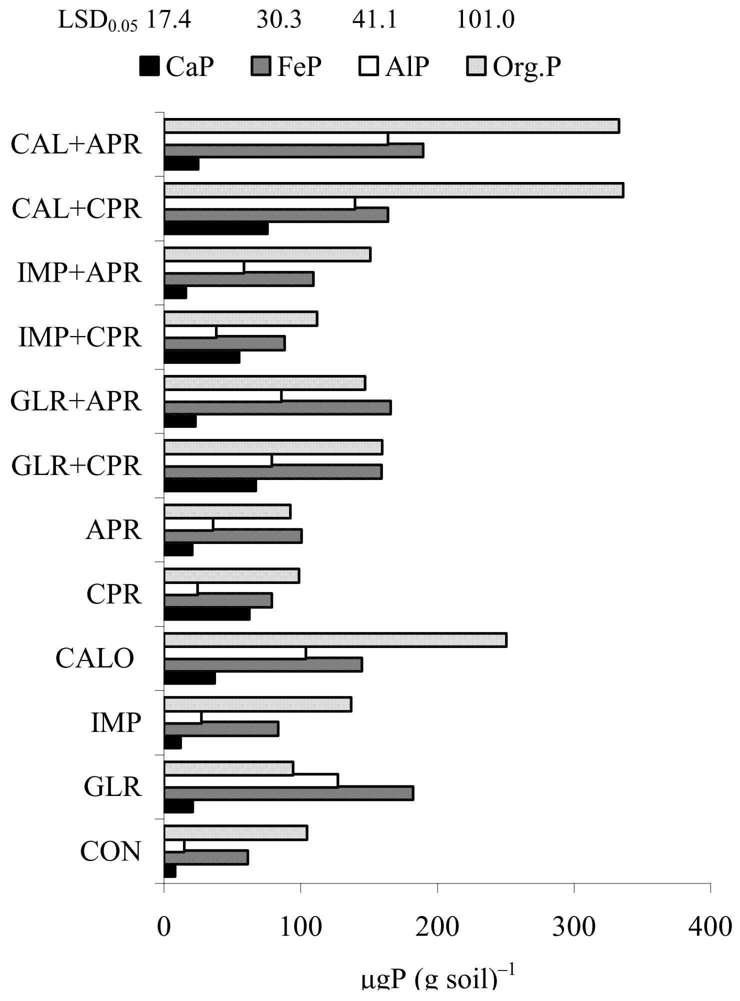

**FIGURE 7.** Fractions of P in a tropical soil treated with GMs and PRs after 16 months of incubation.

In the second phase (>4 months), Olsen P increased significantly and more than 20% of GM-P from the legume GMs was available after 8 months. This increased further at the end of the study to about 40–60% of legume GM-P added (Figs. 5 and 6). The change in available P in the second phase was related to GM quality (C-to-P ratio). Thus Calopogonium (C:P = 149) and Gliricidia (C:P = 225) increased available P by a much greater proportion of their organic P content than the poor-quality Imperata GM (0.08% P; C:P = 500). Similarly, the overall effect of the legume GMs was more considerable than that of the P fertilizer (3–4 times after 16 months). In the GM+P mixtures, Calopogonium made much greater improvements in available P than Gliricidia, irrespective of the fertilizer. This was likely due to high Ca release from the latter (Fig. 4). Combinations of NCPR and TSP had similar effect as those of APR (data not shown).

### Soil Nutrient Status

The legume GMs alone and their combinations with P markedly improved the nutrient status of the tropical soil at the end of the incubation (Fig. 7). Even though the pH in the end was less than the initial value, exchangeable K, Ca, and Mg as well as plant available P (Pi) were significantly increased. Gliricidia more than doubled exchangeable Ca, increased exchangeable Mg about 7 times, and K more than 70 times. On the other hand, Calopogonium had little effect on exchangeable Ca in the P treatments, but made significant improvements in the other cation levels (>4 times for Mg and >70 times for K) (Fig. 8). The effect of Imperata was also significant, but much lower compared to the legume GMs. Plant-available P was also augmented at the end of the incubation study because the P released in the earlier stages of the incubation saturated P sorption sites in the soil. This translated into marked increases in the Al-P and Fe-P fractions (Fig. 7). As these two fractions made the most important contributions to plant available P (Table 4), it suggests that that there was very little occlusion of the sorbed P in the presence of the legume GMs[42]. The P fertilizers were less effective in increasing the availability of P than the GMs, which suggests that much of the P released from these materials was irreversibly adsorbed. Nevertheless, the GMs increased the effectiveness of the P fertilizers by 24–30% (Imperata), 40–50% (Gliricidia), and 150–440% (Calopogonium) (Fig. 8). Hence the GMs enhanced the residual effect of the P fertilizers.

The nutrient uptake pattern of *Setaria sphacelata* in the glasshouse experiment also reflected the improved nutrient status of the soil following treatment with the GMs and their mixtures with P fertilizers. These materials markedly increased the uptake of N, P, K, Ca, and Mg. (Figs. 9–11). The independent effect of the GMs was much greater than that of the P fertilizers and there was significant positive interaction between the GMs and P fertilizers in the uptake of P, K, and Mg (Figs. 9 and 11). The GMs considerably improved uptake of P (Fig. 9) as well as that of K and Mg in the fertilizers treatments (Fig. 11). Surprisingly, the low-quality Imperata made comparable or much greater improvements in P uptake from TSP and APR as the legume GMs (Fig. 9). Perhaps the release of high levels of Ca limited the effectiveness of the legume GMs in the GM+P mixtures (especially Gliricidia) by suppressing the dissolution of the PRs. Moreover, the lower content of N, Ca, and Mg in Imperata resulted in net production of acidity during decomposition and this could have promoted the dissolution of the PR[13]. However, the action of Imperata was more to maintain P in available forms rather than the promotion of PR degradation. This may have been achieved in this case by the action of organic acids and possibly the conservation of soil moisture because of the low rate of decomposition of Imperata[42]. This is evidenced by the fact that Imperata enhanced P uptake from highly reactive APR and water-soluble TSP, but not from the poorly reactive CPR (Fig. 9). The highly reactive APR and TSP released more P which was maintained in available forms by Imperata, while poorly reactive CPR released very little P in the first place. On the other hand, the enhancing effect of the legume GMs on CPR was possibly due to the immobilization of Al and Fe in this PR since pH was elevated by the release of $NH_4$-N from these GMs. However, the concomitant release of divalent cations from legume GMs suppressed further PR dissolution and reduced mobilization of adsorbed P.

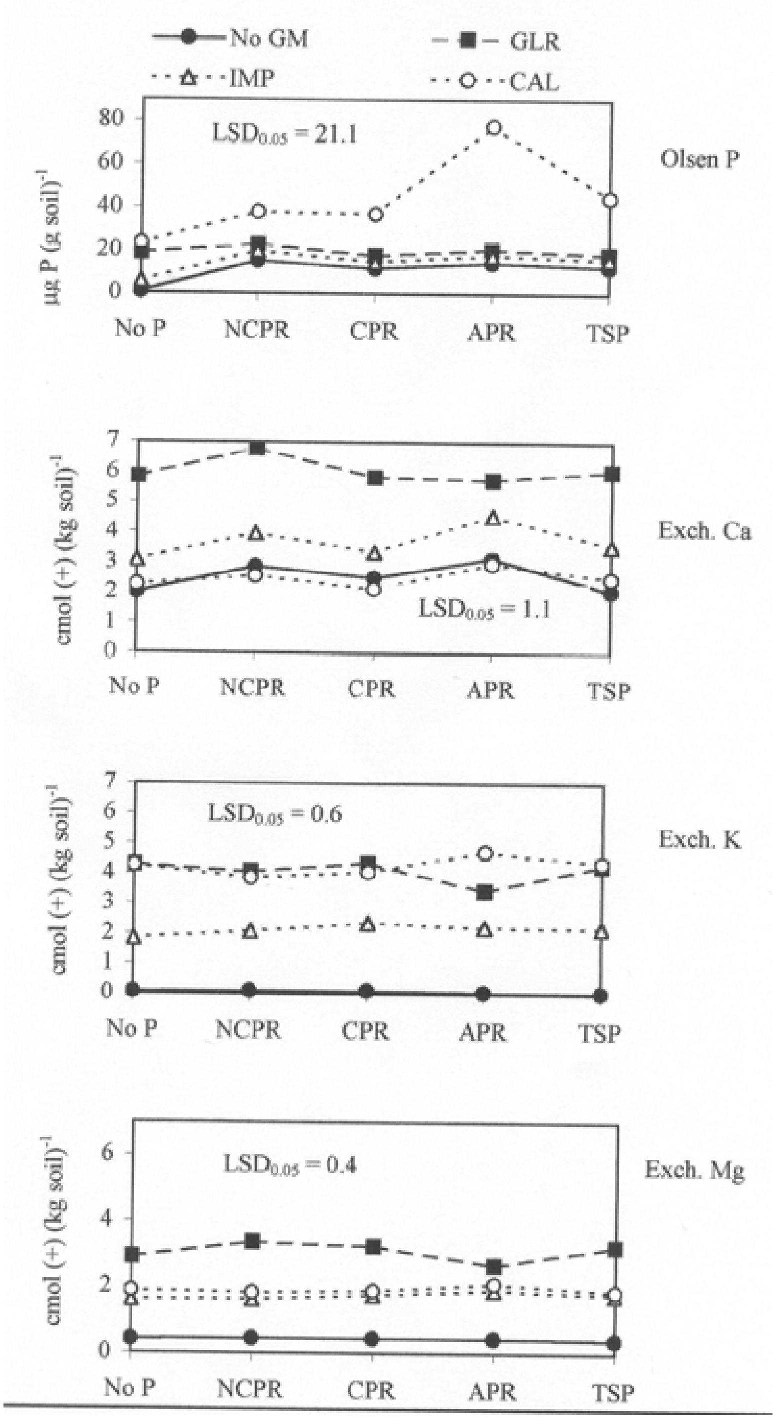

**FIGURE 8**. Nutrient levels in a tropical soil treated with GMs and inorganic P fertilizers after incubation for 64 weeks.

**TABLE 4**
**Stepwise Multiple Regressions Between Soil P Fractions and Available P after a 64-Week Incubation of a Tropical Soil Treated with GMs and P Fertilizers**

| P Fractions | Regression Coefficient, $R^2$ | |
|---|---|---|
| | Olsen P | Pi-P[a] |
| Ca-P | 0.01 | 0.03 |
| Organic-P | 0.46 | 0.59 |
| Fe-P | 0.75 | 0.56 |
| Al-P | 0.83 | 0.70 |
| Ca-P + Al-P | 0.84 | 0.70 |
| Al-P + Fe-P | 0.84 | 0.70 |
| Al-P + Organic-P | 0.83 | 0.75 |
| Fe-P + Organic-P | 0.80 | 0.74 |
| Ca-P + Al-P + Fe-P | 0.85 | 0.70 |
| Ca-P + Al-P + Organic-P | 0.85 | 0.75 |
| Al-P + Fe-P + Organic-P | 0.84 | 0.76 |
| Ca-P + Fe-P + Organic-P | 0.82 | 0.74 |
| Ca-P + Al-P + Fe-P + Organic-P | 0.86 | 0.76 |

[a] Plant-available P.

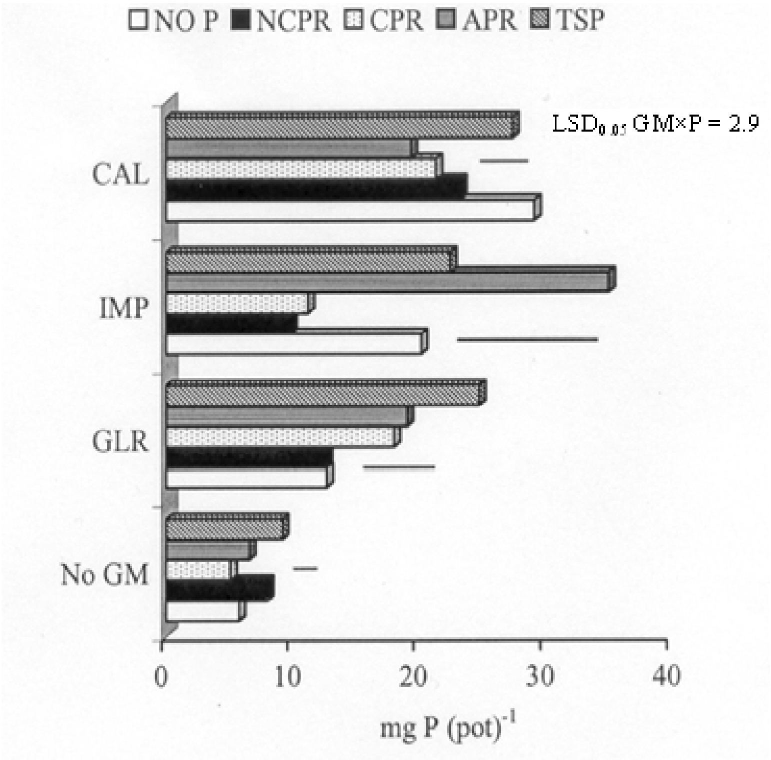

**FIGURE 9.** Total P uptake by *Setaria sphacelata* after 20 weeks growth in a tropical soil as affected by GMs and P fertilizers. Horizontal lines = $LSD_{0.05}$.

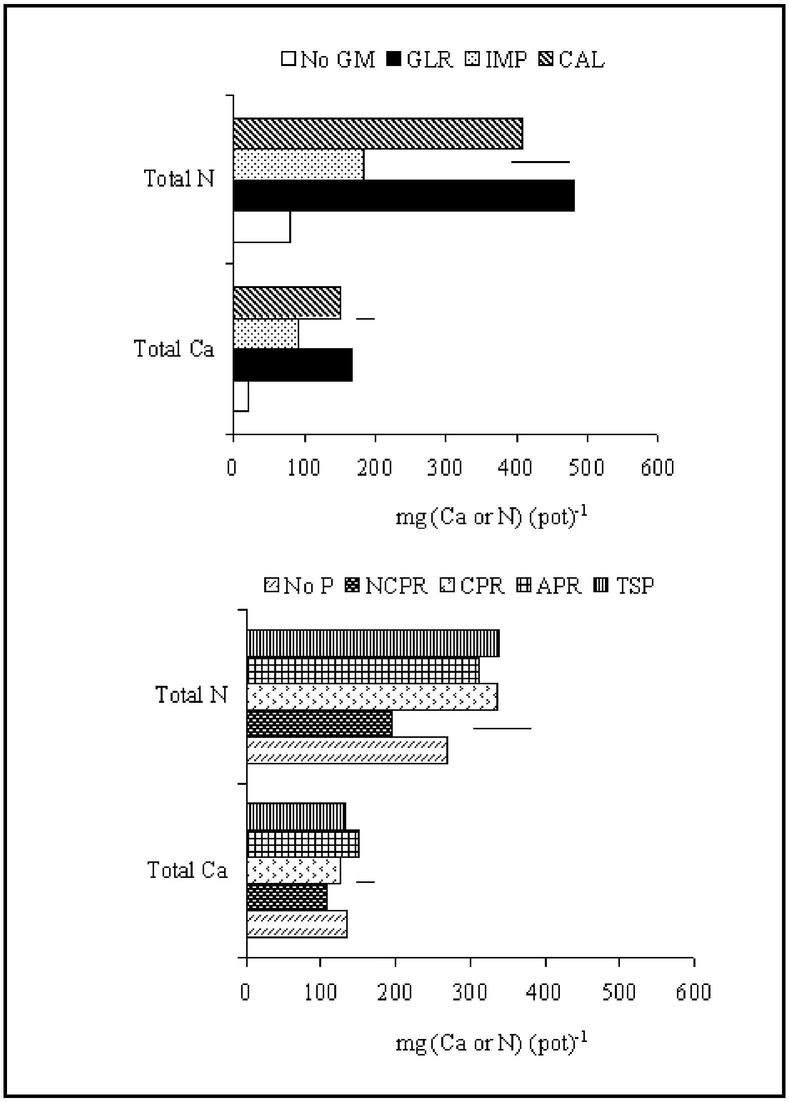

**FIGURE 10.** Uptake of N and Ca by *Setaria sphacelata* after 20 weeks growth in a tropical soil treated with GMs and P fertilizers. Horizontal lines = $LSD_{0.05}$.

### The Potential for Nutrient Management in Tropical Soils

The studies reported here were conducted under controlled conditions, so extrapolation of the results to field situations should be done with utmost caution because environmental factors such as temperature and rainfall can alter the trends described above. Higher temperatures increase GM decomposition rate and hence N mineralization, which could result in greater loss of the released $NH_4$-N through volatilization. Similarly, high rainfall could lead to excessive leaching of released $NO_3$-N. Nevertheless, it is evident that the integration of GMs and PRs offers potential for ameliorating deficiency in N, P, and exchangeable cations as well as eliminating Al toxicity (at least temporarily) in tropical soils. Between 50–60% of N applied as Calopogonium was released as $NH_4$-N. This increased soil ammonium N over a thousand fold and elevated soil pH from 4.5–6.8 within 8 weeks (2 months). This increased P retention initially, but this was reversed after 4 months to increase available P as much as 6 times by the end of the study. Also, the effectiveness of CPR for increasing available P was increased 4–5 times and that of APR 5–7 times by Calopogonium. But the effect of Gliricidia on the PRs was limited by its high Ca content and the low soil CEC.

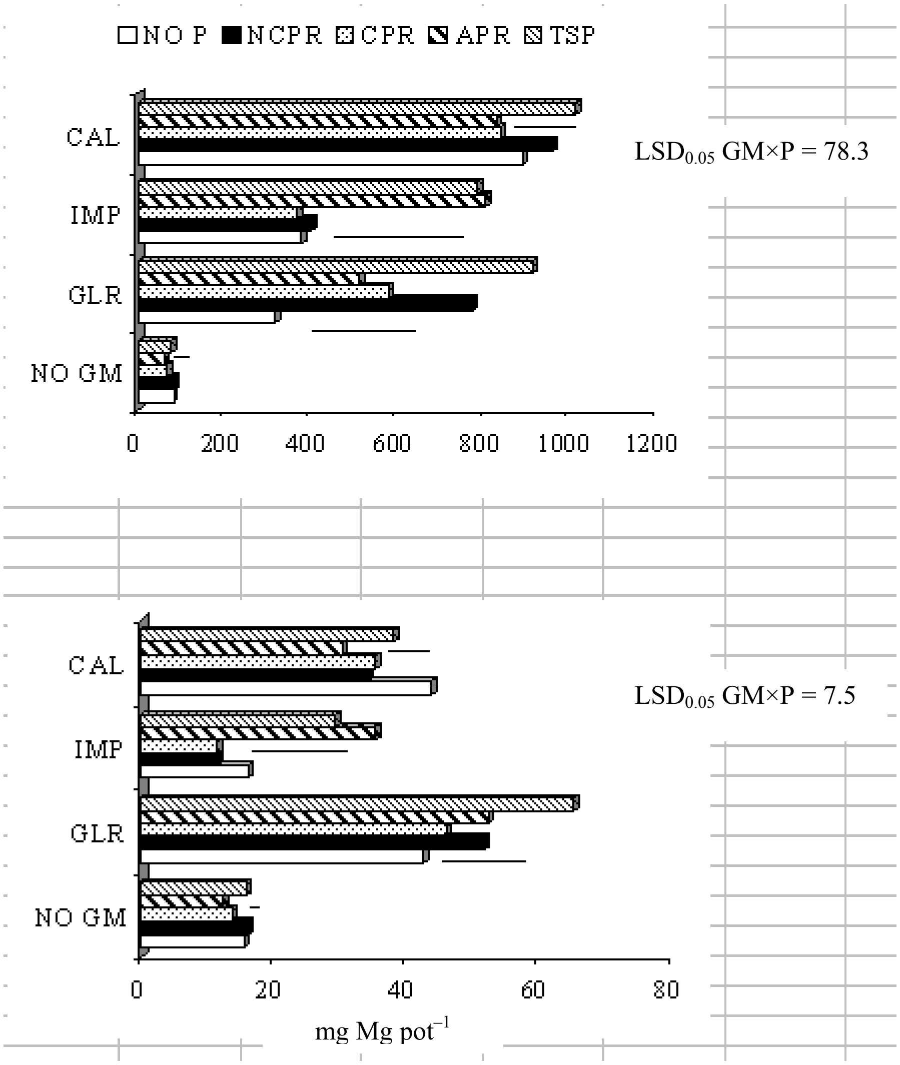

**FIGURE 11.** Uptake of K and Mg by *Setaria sphacelata* after 20 weeks growth in a tropical soil as affected by GMs and P fertilizers. Horizontal lines = $LSD_{0.05}$.

**TABLE 5**
**Percent of total P uptake derived from $^{33}$P labeled GMs by *Setaria sphacelata* grown for 20 weeks in a tropical soil treated with GMs and P fertilizers.**

| GMs | P Fertilizers | | | | |
|---|---|---|---|---|---|
| | NO P | NCPR | CPR | APR | TSP |
| Gliricidia | 4.16a | 1.18a | 1.38a | 1.29a | 0.88a |
| Imperata | 0.64c | 0.30b | 0.62a | 0.34b | 0.68a |
| Calopogonium | 1.51b | 1.16a | 1.31a | 1.21a | 0.72a |
| P×GM | ** | 0.14 | | | |

[1]Values within a column followed by the same letter are not significantly different ($P$< 0.05).

The positive influence of the GMs was also shown in the improvement of the utilization of fertilizer-P. The direct contribution of the GMs to P uptake, measured using $^{33}P$ tracer, was less than 5% (Table 5). However, indirect measurements with $^{32}P$ radioisotope showed that the GMs markedly increased the uptake of fertilizer-P by 40 to over 80% irrespective of GM quality. Calopogonium made the greatest improvement on the uptake of P from NCPR, CPR, and TSP and Imperata had a greater effect on the PR treatments than Gliricidia (Fig. 12). Despite these positive interactions between GMs and P fertilizers, nutrient accumulation patterns in this study suggest that management of these materials may have to be timely and targeted to specific soil and climatic conditions for optimum benefits to be realized. Soils with higher CEC and leaching in high rainfall areas could provide sinks for Ca and increase available P in GM+PR mixtures even in early stages. Since the legume GMs decompose rapidly and can lose large amounts of N through volatilization, management techniques such as split application of the GMs or mixing legume and nonlegume GMs should be considered for minimizing these losses. Other studies have shown that incorporating high-quality organic materials (legume GMs) with those of a lower quality (materials with wide C:N, high lignin, and polyphenol) results in greater immobilization of released N, and hence minimize N loss[43]. Also, applying the GMs after the inorganic P fertilizers would probably improve fertilizer-P availability at later stages. Even though significant levels of fertilizer-P could still be adsorbed initially, much of this was shown to be exchangeable adsorption wherein adsorbed P resided in the Al-P and Fe-P soil fractions (Fig. 7; Table 4). Therefore, the adsorbed P would be made available later on through the action of organic anions released by decomposing GMs added after the P fertilizers[8,14]. This process is continued in the succeeding applications of the remaining GM portions. Such a system could fit in very well with the initial massive input of fertilizer P, designed to saturate sorption sites and enhance availability, in the P capitalization strategy proposed for highly weathered soils in sub-Saharan Africa[6,44]. Thus the legume GMs would serve to improve the availability of immobilized P in this case. However, careful consideration should be given to the choice of compatible GMs and PRs in these integrated systems. The GMs with high Ca contents like Gliricidia in our study may be targeted for soils with high CEC or in high rainfall areas where Ca could be leached out or possibly include plant genotypes with greater affinity for this nutrient. All this would further enhance plant-available P in the treated soils. In addition to N and P, this system can also improve the level of other macronutrients such as K, Ca, and Mg.

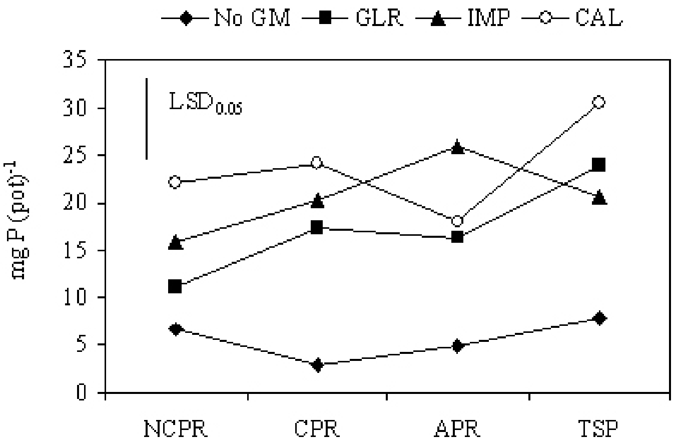

**FIGURE 12.** Total fertilizer-P uptake (indirectly quantified with $^{32}P$) by *Setaria sphacelata* grown for 20 weeks in a tropical soil treated with inorganic P fertilizers and GMs.

### CONCLUSIONS

The benefit of integrating GMs and inorganic P fertilizers for combating acid soil infertility in the tropics was demonstrated in this investigation. The legume GMs increased soil pH to over 6.5 (albeit for only 8–16 weeks) and significantly enhanced soil nutrient status (N, P, K, Ca, and Mg). However, the $^{33}$P direct labeling technique showed that the GMs provided very little plant available P (<5%). On the other hand, the indirect method wherein the soil was labeled with $^{32}$P proved that the GMs significantly enhanced the availability of fertilizer P by as much as 40 to over 80%, irrespective of GM quality.

These results have important implications for the sustainable management of highly weathered tropical soils. However, further field evaluation of this system is required where other factors such as leaching and much higher $NH_3$ volatilization could alter nutrient dynamics dramatically. Also, the potential of Imperata, a ubiquitous weed in the tropics, in integrated GM-PR systems including socioeconomic factors needs more in-depth evaluation. This could provide great opportunities for resource poor farmers in these regions who must maximize natural resource use because of precarious economic circumstances and fragile environment.

### ACKNOWLEDGMENTS

We are grateful to the Malaysian Government for financial assistance under the Malaysian Technical Cooperation Programme.

### BIOSKETCHES

**Abdul Rahman Bah, PhD**, is a postdoctoral fellow in the Department of Land management, Faculty of Agriculture at the Universiti Putra Malaysia. His research interests include nutrient cycling, integrated nutrient management and sustainable cropping systems.

**Zaharah Abdul Rahman, DSc,** is a professor in the Department of Land Management at the Universiti Putra Malaysia where she runs courses in soil fertility and soil chemistry. She is an expert on the use of isotopes in soil/plant studies and has been training researchers and technicians from developing countries in collaboration with the International Atomic Energy Agency (IAEA). Her research interests include fertilizer use by crops in different cropping systems.

**Aminuddin Hussin, PhD**, is an Associate professor in the Department of Land Management at the Universiti Putra Malaysia. He is interested in fertiliser use efficiency studies in oil palm, and is currently investigating methods of improving urea use efficiency in oil palm.
